# Decentralising paediatric hearing services through district healthcare screening in Western Cape province, South Africa

**DOI:** 10.4102/phcfm.v13i1.2903

**Published:** 2021-06-29

**Authors:** Silva Kuschke, Talita le Roux, Alex J. Scott, Daniel C. d.W. Swanepoel

**Affiliations:** 1 Department of Audiology, Faculty of Allied Health – Communication Sciences, Red Cross War Memorial Children’s Hospital, Cape Town, South Africa; 2 Department Speech-Language Pathology and Audiology, Faculty of Humanities, University of Pretoria, Pretoria, South Africa; 3 Department of Medicine, Faculty of Health Science, University of Cape Town, Cape Town, South Africa; 4 Department Ear Science Centre, School of Surgery, University of Western Australia, Nedlands, Australia; 5 Ear Science Institute, Subiaco, Australia

**Keywords:** childhood hearing loss, decentralisation, hearing healthcare, low- and middle-income countries, otoacoustic emissions

## Abstract

**Background:**

Childhood hearing loss is a global epidemic most prevalent in low- and middle-income countries where hearing healthcare services are often inaccessible. Referrals for primary care services to central hospitals add to growing lists and delays the time-sensitive treatment of childhood hearing loss.

**Aim:**

To compare a centralised tertiary model of hearing healthcare with a decentralised model through district hearing screening for children in the Western Cape province, South Africa.

**Setting:**

A central paediatric tertiary hospital in Cape Town and a district hospital in the South Peninsula region.

**Methods:**

A pragmatic quasi-experimental study design was used with a 7-month control period at a tertiary hospital (June 2019 to December 2019). Decentralising was measured by attendance rates, travelling distance, number of referrals to the tertiary hospital and hearing outcomes. There were 315 children in the tertiary group and 158 in the district group. Data were collected from patient records and an electronic database at the tertiary hospital.

**Results:**

Attendance rate at the district hospital was significantly higher (*p* < 0.001). Travel distance to the district hospital was significantly shorter (*p* < 0.001). Number of referrals to the tertiary hospital decreased significantly during the intervention period (*p* < 0.001). Most children in both the tertiary and district groups (78.7% and 80.4%, respectively) passed initial hearing screening bilaterally.

**Conclusion:**

Hearing screening should be conducted at the appropriate level of care to increase access, reduce patient travelling distances and associated costs and reduce the burden on tertiary-level hospitals.

## Introduction

Hearing loss is the second most prevalent developmental disability, affecting approximately 15.5 million children under the age of 5 years globally.^1^ Approximately 95% of children with developmental disabilities reside in low- and middle-income countries (LMICs).^1^ Sub-Saharan Africa has one of the highest prevalence rates of hearing loss,^2^ with an estimated 10.3 million children under the age of 10 years who suffer from permanent disabling hearing loss.^3^ Undetected and untreated hearing loss has a major negative impact on a child’s speech, language, cognitive, educational and socio-emotional development.^4^

Hearing healthcare services in LMICs are not prioritised by health systems overwhelmed by life-threatening diseases.^5^ Identification of hearing loss in children is often impeded in LMICs because of the absence of well-managed hearing screening programmes, the impact of poverty and malnutrition on hearing and the lack of public and professional awareness of hearing loss and its devastating effects in children.^6^ In addition, poor hearing health infrastructure and resources (personnel and equipment) and geographical barriers such as distance, lead to limited accessibility of hearing healthcare services.^5,6^ Children born into a lower socioeconomic status have considerably less access to non-emergency health resources.^7,8,9^ Furthermore, the risk of poor follow-up rates for hearing assessments and timely intervention is higher in families who need to travel greater distances.^10,11^

Compared with high-income countries, LMICs have an unequal proportion of hearing loss burden and a limited number of well-trained hearing healthcare professionals.^12^ The number of audiologists and Ear–Nose–Throat (ENT) specialists are reported to be lowest in African countries, with an average estimate of one audiologist for every 0.8 million people and one ENT specialist for every 1.2 million people in sub-Saharan Africa.^13^ Over a 10-year period, between 2005 and 2015, there has been no substantial improvement in these numbers.^13^

In LMICs such as South Africa, healthcare facilities are typically tiered into three main levels of care: primary such as point-of-entry clinics, secondary that includes district and regional hospitals and tertiary which encompasses specialised services.^14^ As a result of the limited number of primary-level hearing screening sites in these settings, children are often referred directly to a centralised tertiary-level hospital for initial hearing screening, when available. Referrals for primary care services such as hearing screening at central tertiary-level hospitals add to growing waiting lists for specialised care such as diagnostic hearing assessments and hearing aid fittings. Direct referrals to a central tertiary hospital often imply that parents and caregivers must travel further to access hearing healthcare infrastructure, which may in turn lead to poor follow-up rates, late diagnoses and late access to hearing technology. Childhood hearing loss impedes speech, language and academic development,^15^ and early auditory stimulation is crucial to minimise the adverse effects of hearing loss in children.^16^

Access to sustainable hearing healthcare services in LMICs is an important public health priority.^17^ Innovative service delivery models, with an emphasis on decentralisation, are required to develop sustainable services in these settings.^18^ Decentralisation is the transfer of responsibility for planning, management and financing from central to peripheral levels of government and has been a key health sector reform in a wide range of LMICs over the past decade.^19^ Despite being implemented as a strategy across many health systems, the impact of decentralisation on health equity is still unclear.^20^ However, it has been suggested that in order to minimise such inequity, government, health sectors and communities must address socio-economic and financial barriers and implement complementary mechanisms alongside decentralisation.^20^

The growing burden of hearing loss in LMICs^21^ is disproportionate to the lack of hearing healthcare services available and current efforts to reach underserved communities are inadequate.^5^ If hearing healthcare services are not available at primary-level healthcare clinics, many communities in LMICs do not have access to these services at all^22^ and tertiary-level services are being overburdened with screening services that should be conducted at a lower level of care. Therefore, approaches that incorporate the delivery of community-based hearing care in order to decentralise hearing healthcare services is a priority.^23,24^

This study aimed to compare a centralised tertiary model of hearing healthcare to a decentralised model through district hearing screening for children in the Western Cape province, South Africa. The effects of a decentralised model of hearing healthcare were measured in terms of attendance rates for initial hearing screening, patient travelling distance, number of referrals to a tertiary-level hospital and hearing outcomes.

## Research methods and design

### Study design

A pragmatic quasi-experimental study design was implemented, with a 7-month control group receiving standard hearing service provision at a tertiary hospital (from June 2018 to December 2018), compared with a 7-month intervention group where hearing screening was offered at a district hospital (from June 2019 to December 2019).

### Setting

The Cape Town metropole has a population of 4 067 774^25^ and is situated in the Southern Peninsula of the Western Cape province, South Africa. The metropole incorporates eight health subdistricts with eight district-level hospitals of which only three have audiology services. Victoria Hospital is a district hospital with 159 beds in the South Peninsula health district of the metropolitan region and currently has no audiology services. No audiological services are available at any of the primary healthcare clinics or maternity and obstetric units (MOU) in this area, which result in referrals for initial hearing screening of older children based on risk factors or concerns for hearing loss. All patients aged 0–13 years who are from the district hospital catchment area and who need audiology services are referred directly to Red Cross War Memorial Children’s Hospital, which is a central tertiary-level hospital in Cape Town.

The Western Cape has three tertiary academic hospitals. Red Cross War Memorial Children’s Hospital is one of two dedicated paediatric tertiary-level academic hospitals in sub-Saharan Africa and serves as a central referral hospital for paediatric patients across the entire Western Cape who require specialised healthcare services. The Department of Audiology at this tertiary facility assesses and provides hearing rehabilitation for approximately 300 children per month. Referrals are received from district hospitals, primary level clinics and MOUs. Both the district and tertiary hospitals in this study are situated in a LMIC and serve mostly children from the public healthcare sector who do not have access to private medical insurance.

### Study population and sampling strategy

Consecutive sampling was used to select participants for both the tertiary and district groups.

#### Tertiary group sampling

All patients who were referred to the tertiary hospital via email for initial hearing screening from the district hospital catchment area during the control period (June 2018 to December 2018), and who attended their hearing screening appointment at the tertiary hospital, were included in the tertiary group, regardless of the reason for referral. These patients were retrospectively selected from the audiology departmental electronic database at the tertiary hospital to form the tertiary group of 315 paediatric patients.

#### District group sampling

All consecutive referrals for initial hearing screening from facilities that fell within the district hospital catchment area were sent via email to the tertiary hospital during the intervention period (from June 2019 to December 2019). These referrals were selected for the decentralised hearing screening project at the district hospital. Only referrals who met the specified inclusion criteria for the district hearing screening project were included in the district group. The primary method of hearing screening for the district group utilised otoacoustic emissions (OAEs), which assesses cochlear function, therefore, referrals for initial screening of high-risk patients who presented with risk factors for retro-cochlear pathology or auditory neuropathy spectrum disorder (e.g. prematurity < 34 weeks gestation, low birthweight, hyperbilirubinaemia and congenital syndromes associated with hearing loss) were excluded and booked at the tertiary hospital. Patients with known middle ear pathology such as otitis media or otorrhoea were also excluded from the district group, as they were likely to fail screening because of middle ear abnormality and would have been better served at the tertiary hospital with a diagnostic hearing assessment.

As a result of limited time and space available at the district hospital, only 10–15 paediatric patients were booked per afternoon twice per month for the 7-month intervention period, which equated to a sample size of 190 referred patients. Parents of referred children were contacted telephonically by the tertiary hospital’s audiology clerk to arrange an appointment for a hearing screening at the district hospital during the intervention period (from June 2019 to December 2019). Children who attended their initial hearing screening appointment at the district hospital were included and formed the district group of 158 patients. The hearing screening at the district hospital was conducted by two audiologists from the tertiary hospital. Most of the hearing screening appointments coincided with routine follow-up paediatrician visits at the district hospital.

#### Data collection

An electronic patient database from the Department of Audiology at the tertiary hospital was used to retrospectively review data of the patients from the district hospital catchment area who were referred to the tertiary hospital for initial hearing screening during the control period (from June 2018 to December 2018). Data included demographic information, reason for referral, initial hearing screening results and number of children from the district hospital catchment area who were referred directly to the tertiary hospital. Only initial OAE hearing screening results were included for the tertiary group, as diagnostic testing was carried out on the same day at the tertiary hospital if a patient referred OAE screening unilaterally or bilaterally, instead of scheduling a rescreen 2 weeks later at the tertiary hospital. Diagnostic assessment results were also included for those children who referred initial OAE screening unilaterally or bilaterally in the tertiary group. The same electronic patient database was used to review the number of children from the district hospital catchment area who were referred to the tertiary hospital for initial hearing screening during the 7-month intervention period at the district hospital (from June 2019 to December 2019).

A hearing screening data sheet for the 7-month intervention period at the district hospital (from June to December 2019) was used to record patient data in terms of demographics, geographical area of residence, reason for referral, OAE screening results and need for further diagnostic testing. Patients in the district group who referred the initial screening unilaterally or bilaterally underwent tympanometry to check their middle ear status and were referred to the paediatrician at the district hospital on the same day as the initial hearing screening in order to treat any middle ear pathology. These patients were rescreened at the district hospital after 2 weeks, and if another unilateral or bilateral refer result was obtained on the rescreen, they were referred for diagnostic hearing assessment at the tertiary hospital.

#### Equipment

The Maico Eroscan® OAE test system was used for initial hearing screening during both the control and intervention periods. The system incorporates a screening function with a four-frequency (2000 hertz [Hz] – 5000 Hz) low-to-high distortion-product OAE testing protocol and conducts a fast, automatic test showing a pass or refer result. The signal-to-noise ratio is set at 6 decibels [dB], and a pass result is obtained if three frequencies pass. The reliability and validity of OAEs for use in a screening setting are well-established.^4,10^

#### Data analysis

Data were entered into Microsoft Excel 2016 (Microsoft Corp, Washington) and descriptive analysis was performed. Data were imported into the Statistical Package for the Social Sciences (SPSS) (version 26.0. New York, IBM Corp.) for inferential analysis. Pearson’s Chi-square test was utilised for categorical data, whereas Student’s *t*-test was utilised for parametrical numerical data. A *p*-value of ≤ 0.05 was considered significant.

### Ethical considerations

The study was approved by the University of Pretoria Research Ethics Committee of the Faculty of Humanities (HUM024/0419), the University of Cape Town Human Research Ethics Committee (365/2019), Red Cross War Memorial Children’s Hospital Ethics Committee (RCC203) and the Western Cape Health Research sub-directorate (WC_201906_023). The tertiary hospital in this study has an Outreach Policy Agreement with all Western Cape Health Facilities, which was used in conjunction with a letter requesting institutional permission from the district hospital to conduct an outreach OAE-screening service there twice per month for 7 months. A letter of informed consent was issued to the caregivers of participants prior to data collection. Informed assent was obtained from children over the age of 7 years.

## Results

### Demographics

The mean age of patients at the time of initial hearing screening was 48.4 months (39.0 standard deviation [s.d.]; range: 1–156) and 52.3 months (35.1 s.d.; range: 1–144) in the tertiary and district groups, respectively. The tertiary and district groups were similar in terms of age, gender and language distribution (Table 1).

**TABLE 1:** Demographic characteristics of paediatric patients in the tertiary and district groups.

Demographic information	Tertiary group (*n* = 315)			District group (*n* = 158)			*p*
	*n*	%	s.d. range	*n*	%	s.d. range	
**Mean age in months**	48.4	39.0	1–156	52.3	35.1	1–144	0.287
**Gender**						
Female	121	38.4	-	60	38.0	-	0.801
Male	194	61.6	-	98	62.0	-	-
**Home language**						
English	176	55.9	-	76	48.1	-	-
Afrikaans	64	20.3	-	39	24.7	-	0.192
isiXhosa	50	15.9	-	34	21.5	-	-
Other	25	7.9	-	9	5.7	-	-

s.d., standard deviation.

### Attendance rates

An attendance rate of 83.2% (158/190) was found during the 7-month intervention period for patients attending the district hearing screening project, which was significantly higher than the attendance rate of 70.2% (315/449) for patients from the district hospital catchment area who were seen for initial hearing screening at the tertiary hospital during the control period (*p* < 0.001).

### Travel distance

The mean travel distance for patients in the district group commuting from home to the district hospital was 12.6 km (7.7 s.d.; range: 1.2–36.8). This distance was significantly shorter than the travel distance of 19.1 km (9.1 s.d.; range: 5.1–37.6), which patients would have had to travel from home to the tertiary hospital (*p* < 0.001).

### Number of initial hearing screening referrals to the tertiary hospital

A total of 1729 patients were referred from facilities across the Western Cape to the tertiary hospital during the control period (from June 2018 to December 2018), of which 449 (26.0%) referrals were for initial hearing screening from the district hospital catchment area. Throughout the intervention period (from June 2019 to December 2019), during which the district screening project was being conducted, the tertiary hospital received a total of 1601 referrals from facilities across the Western Cape province, with a significant decrease to 114 (7.1%) referrals for initial hearing screening from the district hospital catchment area (*p* < 0.001).

### Reasons for referral

**FIGURE 1:**
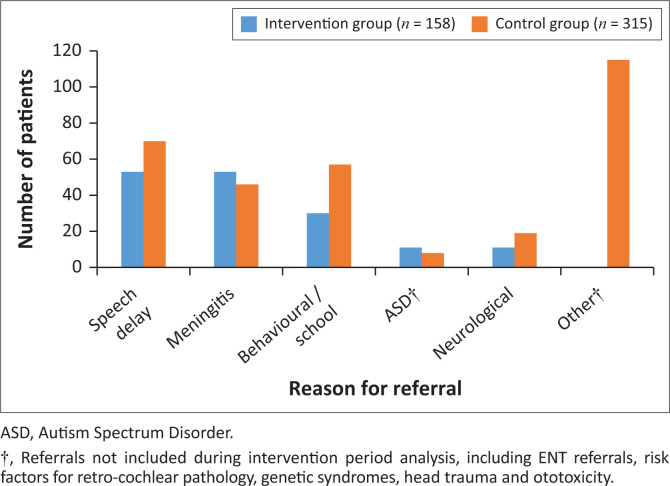
Reason for referral for initial hearing screening.

The reasons for referral for initial hearing screening are depicted (Figure 1). During the control period (*n* = 315), 115 referrals (36.5%) were received for reasons that were excluded from the intervention period analysis. When excluding these 115 referrals, the most common reasons for referral in the tertiary group were speech delay (35.0%) and behavioural or school-related concerns (28.5%) (*n* = 200). In the district group, speech delay (33.5%) and meningitis (33.5%) were the most common reasons for referral (*n* = 158).

### Hearing screening outcomes for the control and intervention period

Outcomes of the initial OAE hearing screenings for the tertiary group and diagnostic assessment results for patients who referred initial OAE screening unilaterally or bilaterally from June 2018 to December 2018 are presented (Table 2). For the tertiary group, most patients (*n* = 248/315, 78.7%) passed the initial OAE screening bilaterally. The number of patients who required diagnostic assessment in the tertiary group were 67 (21.3%). Of the 67 patients who required diagnostic assessment, 54 (80.6%) attended their appointments. Half of the patients (*n* = 27/54, 50%) were diagnosed with mild conductive hearing loss.

**TABLE 2:** Hearing screening outcomes and diagnostic assessment results for the tertiary and district groups.

Demographic information	Tertiary group		District group	
	*n*‡	%	*n*§	%
**Hearing screening outcomes**				
Initial OAE screen				
Bilateral pass	248	78.7	127	80.4
Bilateral refer	41	13.0	15	9.5
Unilateral refer	15	4.8	11	7.0
Bilateral could not elicit	11	3.5	5	3.2
OAE rescreen¶				
Bilateral pass	-	-	11	52.4
Bilateral refer	-	-	6	28.6
Unilateral refer	-	-	4	19.0
**Diagnostic assessment results at the tertiary hospital††, ‡‡**				
Normal hearing	17	31.4	3	27.3
Degrees of hearing loss†				
Mild (21 dBHL – 40 dBHL)				
Conductive hearing loss	27	50.0	5	45.5
Sensorineural hearing loss	0	0.0	0	0.0
Moderate (41 dBHL – 60 dBHL)				
Conductive hearing loss	4	7.4	1	9.1
Sensorineural hearing loss	3	5.6	1	9.1
Profound (> 80 dBHL)				
Conductive hearing loss	0	0.0	0	0.0
Sensorineural hearing loss	3	5.6	1	9.1

Hz, hertz; dBHL, decibel hearing level; OAE, otoacoustic emissions.

† , Pure tone average threshold for worst ear across 500 Hz, 1000 Hz and 2000 Hz.

‡ , *n* = 315.

§ , *n* = 158.

¶ , District group: *n* = 21/26.

†† , Tertiary group: *n* = 54/67.

‡‡ , District group: *n* = 11/15.

Outcomes of the initial OAE screenings from the intervention period at the district hospital and the diagnostic assessment results for patients referred to the tertiary hospital after a unilateral or bilateral refer result on rescreening at the district hospital, are also presented (Table 2). For the district group, most patients (*n* = 127/158, 80.4%) passed OAE screening bilaterally, whilst less than 10% referred OAE screening in both ears. The follow-up attendance rate for rescreening at the district hospital 2 weeks after the initial screening was 80.8% (*n* = 21/26). The total number of patients in the district group that needed referral to the tertiary hospital for specialised diagnostic assessment were 15 (*n* = 15/158, 9.5%), of which 11 (*n* = 11/15, 73.3%) attended the diagnostic hearing assessment appointment. Of these 11 patients, nearly half (*n* = 5/11, 45.5%) presented with mild conductive hearing loss.

## Discussion

This study explored the effect of decentralising hearing healthcare services from a tertiary-level hospital to a district-level hospital in the Western Cape province, South Africa. Decentralised hearing screening resulted in increased attendance rates for initial hearing screening, shorter travelling distances for patients and decreased referral rates to a tertiary-level hospital.

Attendance rates were significantly higher for initial hearing screening at the district hospital when compared with initial screening at the tertiary hospital. Non-attendance can result in underutilisation of healthcare provider time and can lead to longer appointment waiting time for patients.^26^ Furthermore, especially in severely resource-constrained settings typical of LMICs, non-attendance delays the identification, diagnosis and timeous intervention of healthcare conditions.^27^ The Health Professions Council of South Africa Early Hearing Detection and Intervention Guidelines^28^ suggest that a 70% and higher follow-up return rate for hearing screening is considered ideal, but that the feasibility of attaining a high follow-up rate is influenced by various factors such as access to healthcare facilities and personal constraints such as poverty.^28^

The follow-up attendance rate for rescreening at the district hospital two weeks after the initial screening was high (80.8%). This could be attributed to the fact that the second screening was also conducted at a community level and coincided with a paediatrician visit to follow up on middle ear pathology for the majority of patients who referred OAE screening bilaterally. A high follow-up attendance rate (89.4%) for hearing screening was also found in a recent South African community-based study when the rescreening was conducted at a community-level as opposed to a public healthcare institution.^29^ Patients who needed referral to the tertiary hospital for specialised diagnostic assessment had an attendance rate of 73.3%, which is in line with a previous South African community-based hearing screening study that found an attendance rate for diagnostic assessments of 75.8%.^29^

Patient travelling distance was significantly shorter to the district hospital as opposed to the tertiary hospital. Access to services is one of the leading barriers to hearing healthcare in underserved communities.^12^ The costs involved in attending healthcare appointments, both in terms of time taken off from work and travel costs for patients with limited resources, remain a further challenge in accessing healthcare in LMICs.^30^ Therefore, primary healthcare is an important strategy employed in South Africa, in order to provide more accessible patient-centred services closer to home.^30^ Community delivered hearing healthcare models have been identified as an important strategy to increase the accessibility and affordability of hearing healthcare in underserved communities.^24,31^

The inaccessibility of hearing healthcare services at a primary- or district-level, which adds severe strain on tertiary-level specialised services, may be alleviated by decentralising services. The results of this study corroborate this. The number of direct referrals for initial hearing screening from the district hospital catchment area to the tertiary hospital significantly decreased after implementation of the decentralised hearing screening project at the district hospital. The decreased number of referrals to the tertiary hospital for initial hearing screening support decreased waiting times and improved capacity to provide specialised diagnostic hearing assessments and intervention to patients requiring tertiary-level care.

More than 80% of children who attended the initial hearing screening during the intervention period at the district hospital passed initial OAEs bilaterally. This high pass rate is a positive outcome for the premise of decentralising hearing screening services to a more appropriate level of care. The majority of patients (78.7%) in the tertiary group also passed initial OAE screening, which supports the premise that hearing outcomes are similar for initial hearing screening regardless of the level of care where hearing screening is conducted. Telehealth applications are available for hearing assessment of older children,^24^ however, utilising OAEs in a screening setting is advantageous in terms of time taken to conduct and minimal training that is required.

The referral rate for diagnostic hearing assessment at the tertiary hospital for the children who attended hearing screening during the intervention period at the district hospital was 9.5%. This percentage is higher than the reported referral rate of a South African community-based hearing and vision screening study of 5.4%, which utilised smartphone-based pure tone audiometry screening.^29^ A possible reason for the higher referral rate is the method of screening. Otoacoustic emissions screening is sensitive to middle ear pathology and it is more likely to fail in the presence of abnormal middle ear function.^32^

Referral for diagnostic testing in the tertiary group (21.3%) was twice as high in the district group (9.5%). The higher number of diagnostic assessments in the tertiary group were because of the fact that no opportunity for rescreening after two weeks was provided, as all patients who referred initial screening unilaterally or bilaterally or those for whom OAE screening results could not be elicited, underwent diagnostic assessment on the same day in order to minimise follow-up appointments at the tertiary hospital.

Providing hearing screening at a district level increased access to medical treatment for all children who presented with middle ear pathology as evidenced by abnormal tympanometry results on the day of initial OAE screening. These children were assessed and treated by the paediatrician on the same day, instead of waiting for months to get an ENT appointment at the tertiary hospital. Thus, middle ear pathology was treated timeously and effectively at a more appropriate level of care, decreasing the added burden to long tertiary waiting lists. Early identification of middle ear pathology is a primary-level healthcare service, and it would be more appropriate to refer children even closer to home to their nearest community healthcare centres for treatment.^31^ This would in turn minimise the burden on district level staff and address the problem of preventative hearing loss in children at grassroots level.^31^

A limitation of this study was that tertiary-level audiologists conducted the hearing screening at the district hospital during the intervention period. Future studies should assess the training needs of community healthcare workers and nurses to conduct hearing screening at district hospital facilities. The premise of task-shifting through community-based hearing screening programmes has been proposed as a way to improve access to hearing healthcare.^24,26^ Community healthcare workers and nurses can be trained to screen for hearing loss using mobile health technology via home-based visits to reach vulnerable communities in LMICs,^33^ thereby improving access to hearing healthcare services and reducing the demands on the limited number of hearing healthcare professionals in South Africa.^33^ In addition no sample size calculation was conducted and group size was pragmatically determined by number of patients over the specified time periods.

## Conclusion

Decentralised hearing screening programmes conducted at the appropriate level of care can increase access to hearing healthcare, reduce patient travelling distances and associated costs and reduce the burden on tertiary-level hospitals. Accessible hearing screening yields higher attendance rates, leading to more effective and timeous treatment of the adverse effects of childhood hearing loss.
