# Corrigendum: Decentralising paediatric hearing services through district healthcare screening in Western Cape province, South Africa

**DOI:** 10.4102/phcfm.v14i1.3345

**Published:** 2022-07-01

**Authors:** Silva Kuschke, Talita le Roux, Alex J. Scott, Daniel C. d.W. Swanepoel

**Affiliations:** 1 Department of Audiology, Faculty of Allied Health – Communication Sciences, Red Cross War Memorial Children’s Hospital, Cape Town, South Africa; 2 Department Speech-Language Pathology and Audiology, Faculty of Humanities, University of Pretoria, Pretoria, South Africa; 3 Department of Medicine, Faculty of Health Science, University of Cape Town, Cape Town, South Africa; 4 Department Ear Science Centre, School of Surgery, University of Western Australia, Nedlands, Australia; 5 Ear Science Institute, Subiaco, Australia

In the published article, Kuschke S, Le Roux T, Scott AJ, Swanepoel DCdW. Decentralising paediatric hearing services through district healthcare screening in Western Cape province, South Africa. Afr J Prm Health Care Fam Med. 2021;13(1), a2903. https://doi.org/10.4102/phcfm.v13i1.2903, there was an error regarding the affiliation for Silva Kuschke. As well as having affiliation 1, they should also have affiliation 2, Department of Speech-Language Pathology and Audiology, Faculty of Humanities, University of Pretoria, Pretoria, South Africa.

The authors apologise for this error. The correction does not change the significance of study’s findings or overall interpretation of its results or the scientific conclusions of the article in any way.

